# Genetic diversity of *Plasmodium falciparum* populations in three malaria transmission settings in Madagascar

**DOI:** 10.1186/s12936-021-03776-1

**Published:** 2021-05-27

**Authors:** Fanomezantsoa Ralinoro, Tovonahary Angelo Rakotomanga, Rianasoambolanoro Rakotosaona, Danielle A. Doll Rakoto, Didier Menard, Victor Jeannoda, Arsene Ratsimbasoa

**Affiliations:** 1 National Malaria Control Programme of Madagascar, Androhibe, Antananarivo, Madagascar; 2 Faculty of Sciences, University of Antananarivo, Antananarivo, Madagascar; 3 Centre National d’Application de Recherches Pharmaceutiques, Analamahitsy, Antananarivo, Madagascar; 4 Malaria Genetics and Resistance Unit and INSERM U1201, Institut Pasteur Paris, Paris, France; 5 Faculty of Medicine, University of Fianarantsoa, Fianarantsoa, Madagascar

**Keywords:** *Plasmodium falciparum*, Malaria, Genetic diversity, *msp-1*, *msp-2* gene, Madagascar

## Abstract

**Background:**

Assessment of the genetic diversity of *Plasmodium falciparum* parasites from various malaria transmission settings could help to define tailored local strategies for malaria control and elimination. Such assessments are currently scarce in Madagascar. The study presented here aimed to bridge this gap by investigating the genetic diversity of *P. falciparum* populations in three epidemiological strata (Equatorial, Tropical and Fringes) in Madagascar.

**Methods:**

Two-hundred and sixty-six *P. falciparum* isolates were obtained from patients with uncomplicated malaria enrolled in clinical drug efficacy studies conducted at health centres in Tsaratanana (Equatorial stratum), Antanimbary (Tropical stratum) and Anjoma Ramartina (Fringes) in 2013 and 2016. Parasite DNA was extracted from blood samples collected before anti-malarial treatment. *Plasmodium* species were identified by nested PCR targeting the *18 S rRNA* gene. The genetic profiles of *P. falciparum* parasites were defined by allele-specific nested PCR on the polymorphic regions of the *msp*-*1* and *msp*-*2* genes.

**Results:**

Fifty-eight alleles were detected in the *P. falciparum* samples tested: 18 alleles for *msp-1* and 40 for *msp-2*. K1 (62.9%, 139/221) and FC27 (69.5%, 114/164) were the principal *msp-1* and *msp-2* allele families detected, although the proportions of the *msp-1* and *msp-2* alleles varied significantly between sites. Polyclonal infections were more frequent at sites in the Equatorial stratum (69.8%) than at sites in the Tropical stratum (60.5%) or Fringes (58.1%). Population genetics analyses showed that genetic diversity was similar between sites and that parasite flow within sites was limited.

**Conclusions:**

This study provides recent information about the genetic diversity of *P. falciparum* populations in three transmission strata in Madagascar, and valuable baseline data for further evaluation of the impact of the control measures implemented in Madagascar.

## Background

Falciparum malaria remains a major infectious disease in humans, affecting millions of people in tropical areas, despite the progress of the last decade, achieved principally by scaling up key interventions (vector control measures and better management of malaria cases) [1]. In 2019, the World Health Organization (WHO) recorded 229 million malaria cases, leading to 409,000 deaths, mostly in pregnant women and children under the age of five years living in sub-Saharan Africa (94%) [1].

In Madagascar (total population: 26,969,306 in 2019), malaria is a major public health issue, the fourth leading cause of morbidity in health centres and of mortality in hospitals. In 2019, the incidence of malaria was estimated at 76.1/1,000 inhabitants, with a mean of 2,052,071 cases of malaria (range 1,535,000–2,642,000) and 5,073 deaths from the disease (range 180–9580) annually [1]. These worryingly high figures were due mostly to a significant increase in the number of cases in districts located in high-transmission areas, several malaria outbreaks in the south, and exceptional climatic conditions (cyclones and floods) in recent years [2].

One of the major challenges facing policy makers is the tremendous variability of malaria transmission across Madagascar, depending on regional variations in rainfall, temperature and elevation. The country is typically divided into five epidemiological strata: (i) the Equatorial stratum on the east coast, where malaria transmission is highest and perennial; (ii) the Tropical stratum on the west coast, with seasonal transmission spanning around 6 months (October–April); (iii) the Sub-desert stratum in the south, characterized by a dry and hot climate prone to episodic outbreaks; (iv) the Highland; and, (v) Fringes stratum in the centre of the country, where malaria transmission rates are low and unstable between January and April [3, 4].

The malaria control interventions recommended by the WHO are currently performed free of charge throughout the country by the Malagasy Malaria Control Programme (MMCP). These interventions are based on vector control measures (long-lasting insecticide-treated nets and the indoor spraying of insecticides) and the prompt and effective management of malaria cases detected in health facilities and in the community (i.e., use of rapid diagnostic tests (RDT) for malaria and artemisinin-based combined therapy (ACT) for treatment). No specific strategies for malaria control tailored to the epidemiological context have been designed and implemented locally or regionally. Furthermore, the impact of strategies has been assessed only on the basis of the estimated number of malaria cases recorded by hospital and health centre staff and community workers [4].

The genotyping of *Plasmodium falciparum* parasites has been shown to be a useful tool for exploring genetic diversity (i.e., the complexity and size of the parasite populations) and multiplicity of infection (MOI), i.e., the number of clones per sample, which is generally considered to be strongly correlated with transmission intensity [5–8]. Indeed, parasite genetic diversity and MOI are high in areas with high rates of malaria transmission, whereas they tend to be markedly lower in regions implementing effective malaria control strategies [7, 9]. One of the most widely used techniques for assessing the genetic diversity and MOI of *P. falciparum* is based on the detection, by PCR, of polymorphisms in genes encoding merozoite surface proteins, such as MSP-1 and MSP-2 [10–13]. For MSP-1 (encoded by the *msp-1* gene located on chromosome 9), block 2 is the most polymorphic region, and three families of alleles with polymorphisms of this region have been described (K1, MAD20 and RO33). For MSP-2 (encoded by the *msp-2* gene located on chromosome 2), block 3 is the most polymorphic region, and two families of alleles have been defined on the basis of polymorphisms of this region (FC27 and IC/3D7).

In Madagascar, where malaria transmission rates vary considerably, the routine monitoring of *P. falciparum* genotypes and of the genetic diversity of parasite populations would probably be very useful [14]. However, very little is currently known about the genetic diversity of *P. falciparum* in Madagascar. In this context, the study presented here aimed to provide new data concerning the genetic diversity of *P. falciparum* populations and MOI for malaria parasites isolated from patients with symptomatic malaria in three areas of the island in which the disease is endemic.

## Methods

### Study sites and blood sample collection

**Fig. 1:**
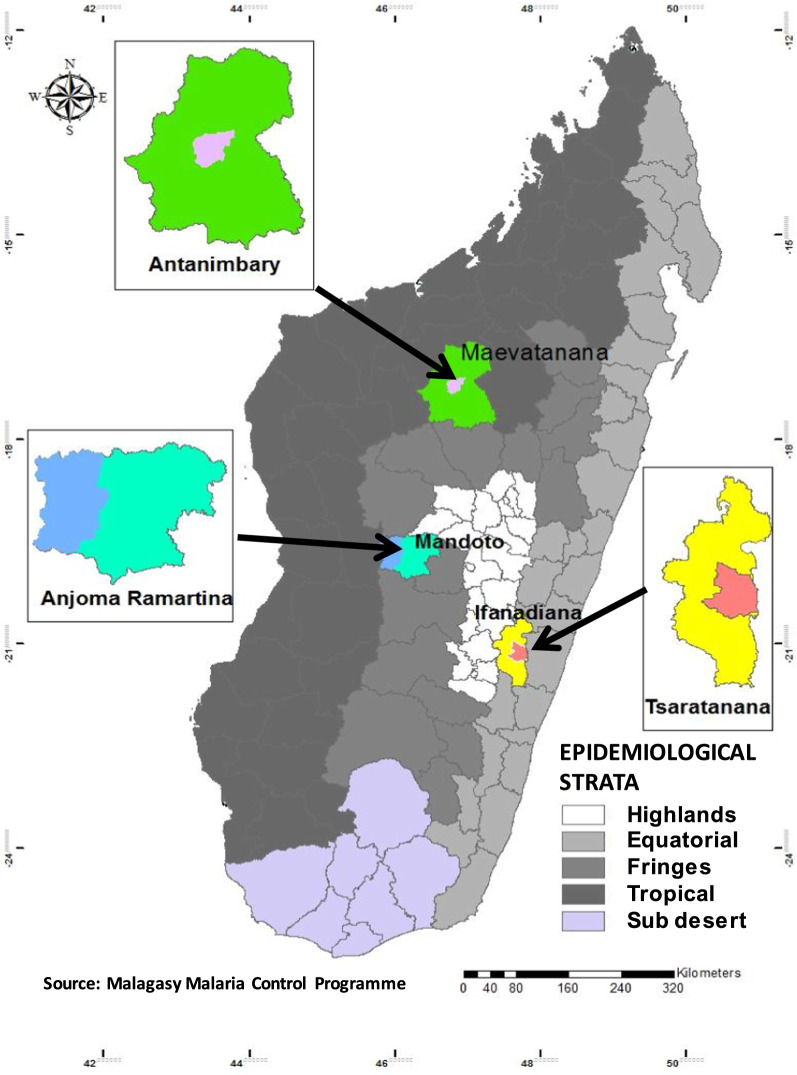
Location of the study sites, according to epidemiological stratum, in Madagascar

Blood samples containing *P. falciparum* were obtained from symptomatic patients presenting at local health centres and enrolled in therapeutic studies evaluating the efficacy of artesunate-amodiaquine treatment in 2013 and 2016 [15]. The patients included were at least six months old, presented uncomplicated falciparum malaria (defined as a positive smear for *P. falciparum* and fever, with body temperature ≥ 37.5 °C) and were resident in one of three zones of endemic malaria in Madagascar: Anjoma Ramartina, a city located in the Fringes, Tsaratanana on the eastern coast (Equatorial stratum) and Antanimbary on the western coast (Tropical stratum) (Fig. 1). Informed consent was obtained from the participants or their parents (for children). Finger-prick blood samples were collected on the day of enrolment, before treatment. The blood samples (100 µL) were used to generate blood films and were spotted onto 3-MM Whatman 903 filter paper (Merck KGaA, Darmstadt, Germany), which were then air-dried and placed individually in plastic bags with desiccant, for transportation to the Malaria Research Laboratory (laboratory of the NMCP) where they were stored at 4 °C until DNA extraction for a maximum of six months.

### Microscopy and parasite counts

Thick and thin blood film slides were stained by incubation with 10% Giemsa solution for 30 min. The stained slides were examined under a light microscope (× 100) for the detection and identification of *Plasmodium* species and for parasite counts by two experienced microscopists. Parasite densities were recorded as the average of the two counts, calculated per 500 white blood cells (WBC), and expressed as the number of parasites/µL of blood, assuming a mean WBC count of 8000/µL of blood. Blood smears with discordant results (differences between the two microscopists in species diagnosis, in parasite density of > 50% or in the presence of parasites) were re-examined by a third, independent microscopist, and parasite density was calculated by averaging the two closest counts [16].

### DNA extraction

Genomic DNA was extracted from the dried blood spots using the QIAamp DNA Blood Mini Kit as per the manufacturer’s instructions (Qiagen, CA, USA) and stored at – 20 °C for further use.

### ***Plasmodium*** species identification and ***msp-1***/***msp-2*** genotyping

Genus- and species-specific nested PCRs targeting the *18 S rRNA* gene were performed, as described by Snounou et al. [10, 17]. The polymorphic regions of the merozoite surface protein genes *msp-1* (block 2), *msp-2* (block 3) were amplified by nested PCR. In the first round of PCR, oligonucleotide primers were used to target conserved genomic regions within *msp-1* (block 2) *msp-2* (block 3). In the second round of PCR, the polymorphic families of *msp-1* (K1, MAD20, and RO33) and *msp-2* (FC27 and 3D7) alleles were amplified with specific primers. The primers and conditions used for first and second rounds of PCR were as described by Oyebola et al. [18]. The PCR products were separated by electrophoresis on a 2% agarose gel, with visualization of the fragments under a gel imager (Gel Doc XR, Biorad) after ethidium bromide staining. The sizes of the alleles (± 20 bp) were determined with molecular weight standards (100 bp DNA Ladder, Invitrogen). DNA from reference *P. falciparum* strains (3D7, Dd2 and 7G8) was included in each run as a control.

### Multiplicity of infection

The MOI or number of genotypes per infection was calculated by dividing the total number of fragments detected for one antigenic marker by the number of samples positive for the marker concerned. The mean MOI was calculated by dividing the total number of fragments detected for both the *msp-1* and *msp-2* loci by the number of samples positive for both markers. Isolates carrying more than one family of alleles were considered to correspond to polyclonal infections, whereas the presence of a single allele family was considered to indicate a monoclonal infection [19].

### Statistical analyses

Statistical analyses were performed with MedCalc version 12 (Mariakerke, Belgium). Mann-Whitney tests were used for non-parametric comparisons and Student’s *t* tests or one-way ANOVA were used for parametric comparisons. For proportions (expressed as percentages), χ^2^ or Fisher’s exact tests were used. *p* values below 0.05 were considered significant. Genetic diversity was assessed by calculating Nei’s unbiased expected heterozygosity (He) from haploid data as follows: *He* = [*n*/(*n *– 1)][1 – pi] (*n* = the number of isolates sampled; pi = the frequency of the itch allele [20]. Population genetic differentiation was assessed with Wright’s F statistic [21]. Population genetic parameters were computed with FSTAT software, v2. 9. 4 [22].

## Results

### Study populations

Two-hundred and sixty-six *P. falciparum* isolates were obtained from patients with uncomplicated malaria seeking anti-malarial treatment at health centres in Anjoma Ramartina (*n* = 85), Tsaratanana (*n* = 79) and Antanimbary (*n* = 102) (Fig. 1). The characteristics of the study populations are described in Table 1.

**Table 1:** Characteristics of the patients enrolled in Anjoma Ramartina, Tsaratanana and Antanimbary

Characteristic	Anjoma Ramartina (Fringes)	Antanimbary (Tropical)	Tsaratanana (Equatorial)	*P*-value
Population size	85	102	79	
Age, years (mean ± SD)	14.2 (10.4)	15.4 (12.8)	6.2 (3.9)	< 0.001*
< 5 years	15	18	32	< 0.001**
5–15 years	39	45	47	
> 15 years	31	39	–	
Gender ratio (male/female)	37/48	41/61	48/31	0.01**
Axillary temperature, °C (mean ± SD)	–	38.2 (1.4)	38.4 (1.0)	NS*
Geometric mean parasitaemia/μL	7,297	16,740	26,282	0.003*

*NS* not significant

*ANOVA, **Chi-squared test

### Frequency and genetic diversity of the ***msp-1*** and ***msp-2*** allele families

**Fig. 2:**
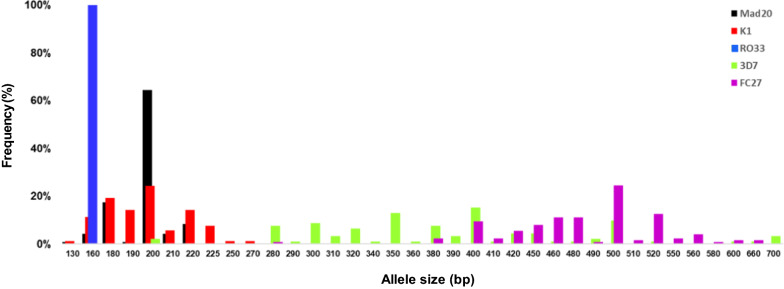
Distribution and proportions of *msp-1* and *msp-2* alleles

Two-hundred and sixty six isolates were genotyped: for 245 isolates (92.1%), amplification was successful for at least one of the two genes, whereas for 138 (56.3%), the targeted regions of both *msp-1* and *msp-2* were amplified. For *msp-1*, 18 alleles were observed (10 K1-types, 7 MAD20-types and one RO33-type) with band sizes of 130–270 bp. The number of alleles detected for *msp-*2 was higher. In total, 40 different *msp-2* alleles were found, 23 belonging to the 3D7 family and 17 to the FC27 family (fragment sizes of 280–700 bp). The proportions of each allele, by allelic family, are presented in Fig. 2.

### ***msp-1*** genotyping

The RO33 allelic family was found to be monomorphic (with an amplified fragment size of 160 bp), and accounted for 36.7% (81/221) of all *msp-1* genotypes. The K1-type alleles predominated, at a frequency of 62.9% (139/221). The most frequent alleles were 200 bp (24%), 180 bp (19%), 220 bp (14%), and 160 bp (11%) long. Two K1-type alleles were present in 15.8% (22/139) of the isolates. MAD20-type alleles were less frequent (53.4%, 118/221). The 200-bp allele was the most frequent (64%), followed by the 180-bp allele (17%) (Fig. 2). The presence of two MAD20 alleles was also observed, but at a low frequency (2.5%, 3/118).

### ***msp-2*** genotyping

**Fig. 3:**
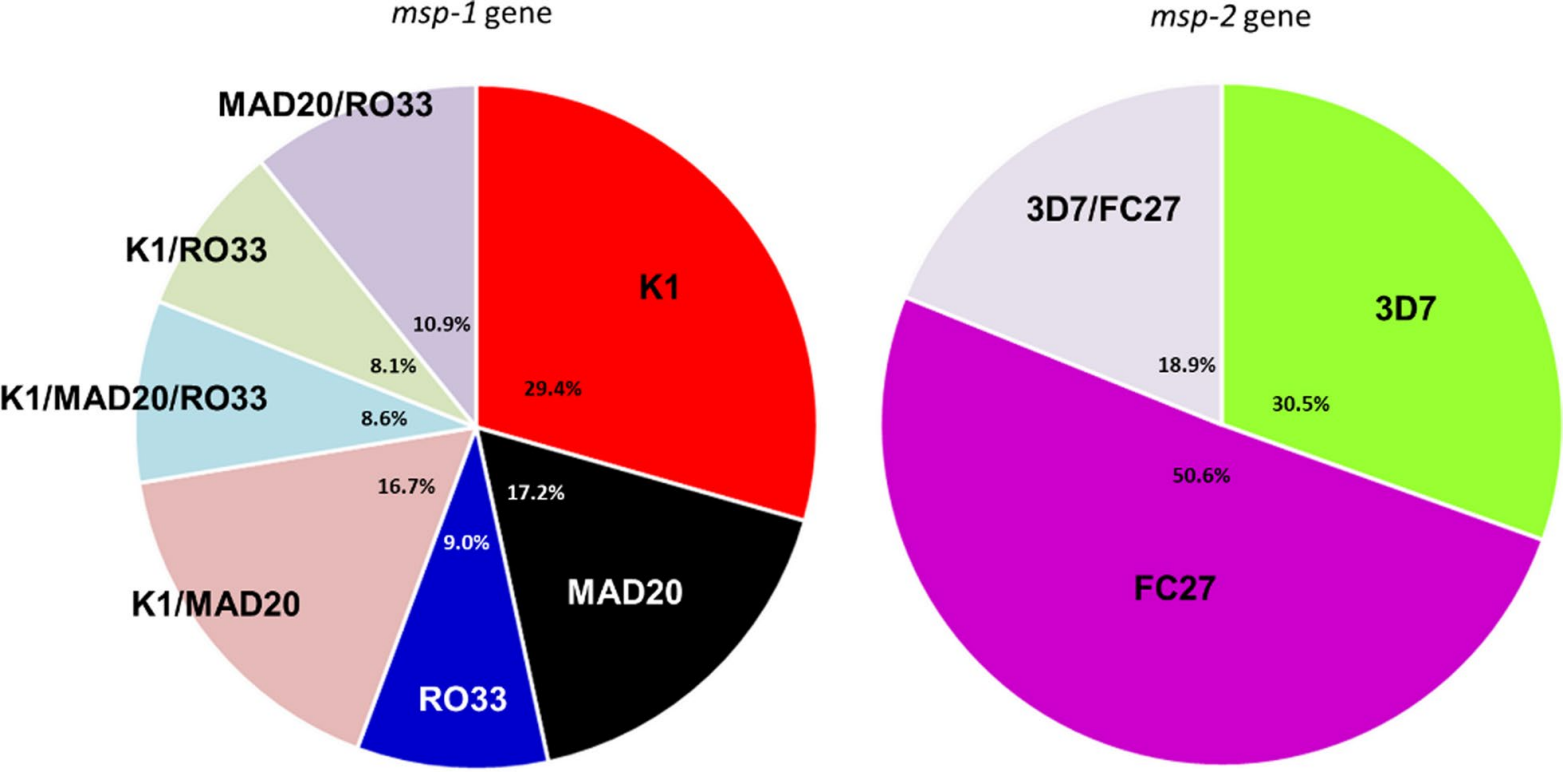
Overall distribution of *msp-1* and *msp-2* genotypes

The 3D7 and FC27 alleles were detected in 49.4% (81/164) and 69.5% (114/164) of *P. falciparum* isolates, respectively. Most of the *msp-2* alleles were observed at low frequency, but seven alleles were more frequent (3D7 allelic family: 400 bp, 15%; 350 bp, 13%; 500 bp, 10%; and, FC27 allelic family: 500 bp, 24%; 520 bp, 13%; 460 bp, 11%; 480 bp, 11%). Several alleles from the same allelic family were present in 13.6% of isolates for the 3D7 family (two alleles in 10/81 and three alleles in 1/81 samples) and in 11.4% for the FC37 family (two alleles). The distribution of the *msp-1* and *msp-2* allelic families is presented in Fig. 3. The proportions of the *msp-1* and *msp-2* alleles varied significantly between the three sites, as shown in Table 2.

**Table 2:** Distribution of *msp-1* and *msp-2* allelic families by study site

Gene	Allelic family	Study site				*P*-value
		Overall	Anjoma Ramartina (Fringes)	Antanimbary (Tropical)	Tsaratanana (Equatorial)	
*msp-1*	*N*	221	67	80	74	–
	K1	29.4%	34.3%	32.5%	21.6%	< 0.0001*
	MAD20	17.2%	7.5%	26.3%	17.6%	
	RO33	9.0%	17.9%	3.8%	6.8%	
	K1 + MAD20	16.7%	1.5%	8.8%	20.3%	
	K1 + RO33	8.1%	7.5%	25.0%	8.1%	
	MAD20 + RO33	10.9%	22.4%	0.0%	12.2%	
	K1 + MAD20 + RO33	8.6%	9.0%	3.8%	13.5%	
*msp-2*	N	164	50	31	83	–
	3D7	30.5%	20.0%	40.7%	30.9%	0.0006*
	FC27	50.6%	67.3%	27.8%	56.4%	
	3D7 + FC27	18.9%	12.7%	31.5%	12.7%	

*N* = population size

*Chi-squared test

### Monoclonal versus polyclonal infections

The proportion of monoclonal infections, as defined by *msp-1* genotyping, was estimated at 55.6% (123/221). The presence of a single allele was most frequent for the K1 allelic family (29.4%), followed by MAD20 (17.2%) and RO33 (9.0%). Combinations of alleles from the RO33, MAD20 and K1 allelic families were detected in 98/221 samples (44.4%). The most frequent combination was K1/MAD20 (16.7%). The proportion of polyclonal infections was significantly higher in Tsaratanana (Equatorial stratum) (60.8%. *p = 0.02*, Chi-squared test) than at the other two sites (40.3 and 42.5%). According to *msp-2* genotyping results, most isolates carried a single allele (monoclonal infection; 81.1%. 133/164). The presence of a single allele was most frequent for the FC27 family (50.6%. 83/164). A combination of alleles from the 3D7 and FC27 allelic families was detected in 31/164 samples (18.9%) (Table 3).

**Table 3:** Proportion of multiclonal infections defined on the basis of *msp-1* and *msp-2* genotyping, by study site, age group and parasite density group

Variable		Multiclonal infections					
		*msp-1*	*P*-value*	*msp-2*	*P*-value*	*Combined msp-1 and msp-2*	P-value*
Overall		106/221 (48.0%)	–	48/164 (29.3%)	–	87/138 (63.0%)	–
By site							
Anjoma Ramartina (Fringes)		27/67 (40.3%)	**0.02**	15/55 (27.3%)	NS	25/43 (58.1%)	NS
Antanimbary (Tropical)		34/80 (42.5%)		21/54 (38.9%)		26/43 (60.5%)	
Tsaratanana (Equatorial)		45/74 (60.8%)		12/55 (21.8%)		36/52 (69.2%)	
By age group							
< 5 years		31/58 (53.4%)	NS	10/41 (24.4%)	NS	24/38 (63.2%)	NS
5–15 years		53/108 (49.1%)		24/86 (27.9%)		44/71 (62.0%)	
> 15 years		22/55 (40.0%)		14/37 (37.8%)		19/29 (65.5%)	
By parasite density group							
< 5000		20/48 (41.7%)	NS	9/39 (23.1%)	NS	19/31 (61.3%)	NS
5000–50,000		54/115 (47.0%)		26/84 (31.0%)		44/70 (62.9%)	
> 50,000		31/57 (54.4%)		12/39 (30.8%)		23/36 (63.9%)	

Significant *P*-value is shown in bold typeface

*Chi-squared test

*NS* not significant

### Population genetic measures: multiplicity of infection, expected heterozygosity and genetic differentiation between sites

The estimated MOI at the three sites is summarized in Table 4. The number of *msp-1* and *msp-2* genotypes per isolate ranged from 1 to 4 and 1 to 3, respectively. The mean MOI per *msp-1* or combined *msp-1* and *msp-2* genotype was significantly higher for isolates from Tsaratanana (Equatorial stratum) (1.92, p = 0.001 and 2.52, p = 0.04, respectively) than for isolates from the other two sites (1.50 and 1.51 and 2.02 and 2.25, for Antanimbary (Tropical stratum) and Anjoma Ramartina (Fringes), respectively). These trends were confirmed in the analysis by age group: the mean MOI (*msp-1* and combined *msp-1/msp-2*) for isolates obtained from patients aged 5–15 years was higher in Tsaratanana (Equatorial stratum) than in Anjoma Ramartina (Fringes) (p = 0.004 and p = 0.007, Mann-Whitney test) and Antanimbary (Tropical stratum) (p = 0.02 for *msp-1*, Mann-Whitney test).

**Table 4:** Multiplicity of infection (MOI) per *msp-1* or *msp-2* and combined *msp-1/msp-2* genotype, by study site, age group and parasite density group

Variable		No. isolates		No. genotypes		MOI (SD)					
		*msp-1*	*msp-2*	*msp-1*	*msp-2*	*msp-* *1*	*P*-value*	*msp-* *2*	*P*-value*	*msp-1/-2*	*P*-value*
Overall		221	164	1–4	1–3	1.64 (0.77)	–	1.34 (0.57)	–	2.28 (1.54)	–
By site											
Anjoma Ramartina (Fringes)		67	55	1–3	1–2	1.51 (0.68)	**0.001**	1.27 (0.45)	NS	2.02 (1.18)	**0.04**
Antanimbary (Tropical)		80	54	1–3	1–3	1.50 (0.63)		1.50 (0.69)		2.25 (1.51)	
Tsaratanana (Equatorial)		74	55	1–4	1–3	1.92 (0.90)		1.25 (0.52)		2.52 (1.81)	
By age group											
< 5 years		58	41	1–4	1–3	1.74 (0.81)	NS	1.27 (0.50)	NS	2.10 (1.08)	NS
5–15 years		108	86	1–4	1–3	1.68 (0.80)		1.34 (0.58)		2.35 (1.75)	
> 15 years		55	37	1–3	1–3	1.47 (0.63)		1.43 (0.60)		2.34 (1.56)	
By parasite density group											
< 5000		48	39	1–3	1–2	1.50 (0.65)	NS	1.23 (0.43)	NS	1.90 (1.07)	NS
5000–50,000		115	84	1–3	1–3	1.63 (0.80)		1.39 (0.64)		2.4 (1.78)	
> 50,000		57	39	1–4	1–3	1.77 (0.80)		1.33 (0.53)		2.33 (1.37)	

Significant *P*-values are shown in bold typeface

*NS* not significant

* ANOVA test

The expected heterozygosity (He) of isolates from the three sites is presented in Table 5. At all sites, He was higher for *msp-2* genotypes (0.823–0.892) than for *msp-1* genotypes (0.413–0.489). However, He was similar between study sites, age groups and parasite density groups.

**Table 5:** Expected heterozygosity (He) estimated by *msp-*1, *msp-2* and combined *msp-1/msp-2* genotyping, by study site

Site	*He (SD)*		
	*msp-1*	*msp-2*	*Combined msp-1/msp-2*
Anjoma Ramartina (Fringes)	0.489 (0.425)	0.892 (0.010)	0.629 (0.363)
Antanimbary (Tropical)	0.440 (0.434)	0.823 (0.129)	0.612 (0.367)
Tsaratanana (Equatorial)	0.413 (0.400)	0.859 (0.04)	0.579 (0.379)

The estimated fixation index (Fst), measuring the population differentiation due to genetic structure at each site, was not significant (Table 6). This finding is consistent with the proportion of genotypes common to the various study sites, as shown in the Table 7. The highest proportion of genotype sharing (6%) was observed between Anjoma Ramartina and Tsaratanana.

**Table 6:** Estimation of the fixation index (Fst) between study sites

Fst	Anjoma Ramartina (Fringes)	Antanimbary (Tropical)
Tsaratanana (Equatorial)	0.04755	0.05824
Anjoma Ramartina (Fringes)		0.05036

**Table 7:** Estimated proportions of *msp-1/-2* genotypes common to different study sites

Sites	Anjoma Ramartina (Fringes) (%)	Antanimbary (Tropical) (%)	Tsaratanana (Equatorial) (%)
Anjoma Ramartina (Fringes)	91	3	6
Antanimbary (Tropical)	3	95	2
Tsaratanana (Equatorial)	6	2	92

## Discussion

Little is currently known about the genetic diversity of *P. falciparum* populations in Madagascar. PCR genotyping analysis with the polymorphic markers *msp-1* and *msp-2* were performed to gain insight into the genetic diversity of the populations of this parasite species in three regions with different patterns of malaria transmission in Madagascar. Excluding genotyping data from clinical trials assessing drug efficacy (performed to distinguish between recrudescence and re-infections in enrolled patients presenting recurrences during follow-up), only two studies, performed in 2000 and 2008, have reported similar analyses [14, 23].

The total number of different *msp-1* and *msp-2* alleles at the three sites (18 and 40, respectively) confirms the high level of malaria transmission in Madagascar. These numbers are similar to those reported in African countries, such as Nigeria, the Republic of Congo, the Central African Republic, Equatorial Guinea and Senegal [24–28].

The predominant alleles were K1-type alleles for *msp-1* and FC27-type alleles for *msp-2*. These findings are consistent with previous reports for Madagascar [14] and other settings in Africa (Nigeria [18, 26], Congo Brazzaville [29], Mauritania [30], Benin [31], Gabon [32, 33], Ivory Coast [34], Cameroon, [35], Ethiopia [36–39]), India [40], and Southeast Asia [41]. However, they contrast with recent reports from Myanmar [42], where MAD20 and 3D7 were the most prevalent alleles.

The distribution of the *msp-1* and *msp-2* allelic families differed significantly between sites (Table 2). Genotyping revealed that half the individuals had *P. falciparum* isolates with a single *msp-1* allele. The proportion of isolates with more than one *msp-1* allele was significantly higher in patients living in the Equatorial stratum, probably reflecting the higher rates of malaria transmission in this setting. This association was confirmed by the significantly higher proportion of polyclonal infections at Tsaratanana (Equatorial stratum, 60.8%) than at the other two sites (40.3 and 42.5%) (Table 3). *msp-2* allelic diversity contrasted more strongly between sites: FC27-type alleles were much more frequent than 3D7-type alleles in the Tropical zone, whereas 3D7-type alleles predominated in the Equatorial and Fringes stratum.

The number of *msp-1* and *msp-2* genotypes per isolate ranged from 1 to 4 and 1–3, respectively. Again, the mean MOI values per *msp-1* or combined *msp-1* and *msp-2* genotype were significantly higher for isolates from patients living in Tsaratanana (Equatorial stratum) (1.92 and 2.52) than for isolates from patients living at the other two sites (1.51 and 2.02, respectively, at Anjoma Ramartina; 1.50 and 2.25, respectively at Antanimbary). These values are similar to those reported in some African countries, such as Ghana [43], Congo Brazzaville [28], and Ethiopia [36–39], but are lower than those reported in Nigeria [26] and Gabon [44]. A similar association was found if the analysis was performed by age group. The mean MOI (*msp-1* and combined *msp-1/msp-2*) of isolates obtained from patients aged 5–15 years was higher for Tsaratanana (Equatorial stratum) than for Anjoma Ramartina (Fringes) and Antanimbary (Tropical stratum). However, no association was found between mean MOI and parasite density, contrary to the findings of several other reports [45].

These data confirm that MOI assessments provide a good assessment of malaria transmission intensity and this metric may be considered a useful tool for evaluating the impact of the vector control measures (long-lasting insecticide-treated bed nets and indoor spraying with insecticides) currently implemented throughout Madagascar. Population genetics analyses revealed no significant difference between sites, age groups and parasite density groups. Furthermore, Fst estimates and the low proportion of genotypes common to different study sites indicated that the circulation of the parasite population between sites remained limited.

This study provides recent data for the genetic diversity of *P. falciparum.* It is, however, subject to several limitations. The principal limitation was the use of the *msp-1* and *msp-2* markers for genotyping, which, like other markers based on DNA fragment size, may decrease estimates of genetic diversity. Nevertheless, *msp-1* and *msp-2* are generally considered to be robust polymorphic markers for which genotyping is straightforward in poorly equipped laboratories, such as those available in Madagascar. A second limitation is that the sample collection sites were not selected specifically for this study, but for a clinical trial assessing the efficacy of artesunate-amodiaquine. This limits the opportunities for data extrapolation. Finally, as no direct measurements of malaria transmission, such as the entomological inoculation rate (EIR), were available for the selected sites, it was not possible to investigate the association between these metrics and genetic diversity.

## Conclusions

Despite several limitations, this study provides recent genetic diversity data for *P. falciparum* isolates collected in three regions with different transmission patterns. The information obtained is valuable for guiding the decisions of policy-makers to improve anti-malaria strategies. The continuous evaluation of these metrics would facilitate evaluations of the control measures implemented in Madagascar. Furthermore, the two markers studied here can be used in investigations of malaria outbreaks, which have been frequent in recent years [46] in the southern areas of the country, which generally have low transmission rates. This approach could be used to determine whether outbreaks are due to the clonal expansion of local or imported *P. falciparum* clones and for the design of more appropriate strategies for preventing outbreaks.

## Data Availability

The data are available from the National Malaria Control Programme of Madagascar.
